# Does Background Music Affect Silent Dining Emotions? An Empirical Study of Restaurants during COVID-19

**DOI:** 10.3390/bs12110434

**Published:** 2022-11-04

**Authors:** Yen-Cheng Chen, Ming-Chen Chiang, Ching-Sung Lee, Pei-Ling Tsui

**Affiliations:** 1 Department of Applied Science of Living, Chinese Culture University, Taipei 11114, Taiwan; 2 Ph.D. Program in Nutrition and Food Science, Fu Jen Catholic University, New Taipei City 242062, Taiwan; 3 Department of Restaurant, Hotel and Institutional Management, Fu Jen Catholic University, New Taipei City 242062, Taiwan; 4 Department of Hospitality Management, National Taitung Junior College, Taitung 95045, Taiwan; 5 Graduate Institute of Technological and Vocational Education, National Taipei University of Technology, Taipei 10608, Taiwan

**Keywords:** music preference, positive emotions, emotions during the dining experience, behavioral response

## Abstract

The music environment of a restaurant is an important factor that affects consumer behaviors during the dining experience, especially silent dining behaviors among people who are not encouraged to talk in the context of COVID-19. This study empirically analyzed the influence of consumers’ background music preferences on their emotions and behavioral responses during their dining experience at a high-end Chinese restaurant. A total of 393 valid samples were obtained through purposive sampling and snowball sampling. The research tools used in this study included a personal background information questionnaire, four Oriental and Western music conditions, a background music preference scale, a scale for evaluating emotions during the dining experience, and a behavioral response scale. The results showed that the subjects preferred the Chinese classical music—the *Butterfly Lovers Concerto*. Background music affected the participants’ emotions during their dining experience, and different background music conditions resulted in significant differences in emotions and behaviors. The consumers’ emotions, during their dining experience significantly predicted their behaviors under all four music conditions. The greatest contribution and value of this study stem from the finding that the background music at a restaurant can arouse specific positive emotions in consumers during their dining experience and thus affect their eating behavior.

## 1. Introduction

Music and health is one of the fastest growing fields in the field of music cognition, a field that covers all aspects of human life [1]. Peng-Li et al. [2] pointed out that background music significantly affects the subjects’ explicit and implicit health behaviors. Following Saarikallio [1], music is a physical and auditory phenomenon that includes acoustic and perceptual characteristics related to time, sound, and volume. These characteristics form the basis of health-related music functions. For example, music elements, such as rhythm, music type, volume, and music quality can have calming, energizing or stimulating effects [3,4,5,6] and can stimulate physical fitness and provide social cooperation behaviors and opportunities for rewards [7].

Chinese cuisine is famous worldwide. With its long history of continuous evolution and development, Taiwanese cuisine has not only inherited the essence of Chinese cuisine but has contributed unique Taiwanese ethnic foods with diverse characteristics that reflect Taiwan’s rich immigrant culture. In addition, local characteristics and diverse ethnic cultures have led to the creation of unique gourmet, high-end Taiwanese restaurants. Therefore, this study focused on the relationship between background music and consumer behavior in high-end Chinese restaurants. Different background music conditions were used in this study as atmospheric stimuli. Whether they had significantly different influences on consumers’ emotions and behavioral responses, during their dining experience at a high-end Chinese restaurant were investigated using the Mehrabian–Russell model [8] as the basic theoretical background. Schmitt [9] stated that in the field of marketing, the creation of situations influences consumers’ perceptions of products. Biswas et al. [10] pointed out that retail ambience, especially background music, is an increasingly important marketing tool for stores and restaurants. Zellner et al. [11] and Peng-Li et al. [2] argued that music conditions affect consumers’ choice of meals. In the current context of the COVID-19 pandemic, people are often discouraged from talking when eating at restaurants; while diners are waiting for their meal and wearing a mask, will the background music of the environment have a positive impact on their emotional experience of dining? This is the important focus of this research.

People’s demands for quality of life have been increasing, and the purpose of dining out is no longer just to eat food; increasingly, it focuses on the perfection of the food and the restaurant environment [12]. Pine and Gilmore [13] pointed out that, as consumers’ concepts of consumption change, they pursue not only tangible goods or intangible services but also an atmospheric experience. Therefore, the consumption value depends not only on the price of the product itself but on a favorable environment that can positively influence consumers’ consumption experiences and emotions [14,15]. Heung and Gu [16] pointed out that the atmospheric design of restaurants directly influences consumer satisfaction and behavior. Most relevant studies have also indicated that since background music impacts consumer responses, restaurant operators should include background music in the overall restaurant design [10,17,18,19,20] and should strive to create an appropriate environment for providing consumers with a comfortable dining experience and to improve the overall dining experience, thereby enhancing consumers’ positive emotions.

In the highly competitive food service industry, making a restaurant stand out and enhancing its competitiveness are important challenges confronting restaurant operators [21]. Restaurant operators need to understand not only the emotional needs of consumers but also the current trends in the industry, and they need to continuously adopt innovations and changes to meet consumer and market demands [22]. This study focuses on whether different background music conditions in high-end Chinese restaurants have different influences on consumers’ behavioral responses and emotions.

## 2. Literature Review and Theoretical Framework

### 2.1. Music and Emotional Regulation

According to Reinoso-Carvalho et al. [23], the emotional response triggered by music is transferred to specific pleasures and senses in some way. For example, when people are listening to music, they perceive beer as tasting sweeter. In addition, noise is part of the eating environment, and it may affect people’s eating experience in different ways [24]. Spence et al. [25] pointed out that the cross-influence of music and noise on food perception and consumer behavior may have an important impact on public health.

Music has been used for emotion regulation [26]. Music can change the ambience of a place and thus impact consumers’ consumption experiences [27]. Spence et al. [28] showed that, compared with the absence of background music, congruent music can significantly enhance the enjoyment of wines. Fiegel et al. [29] pointed out that different genres of background music can change the sense of pleasure generated by food, their study provides new empirical evidence that different musical stimuli can alter sensory perception and acceptance of simultaneously presented foods. Specifically, in the presence of jazz stimuli, the taste pleasure and overall impression of food stimuli were increased. Sunaga [30] found that background music with the same product attributes will make consumers have a positive emotional response.

Areni and Kim [31] and North et al. [32] demonstrated that liquor stores and restaurants that played classical music in the background had higher sales and that their customers chose more expensive commodities. North and Hargreaves [33] and North et al. [34] showed that customers purchased more German wines than French wines when a wine shop played German music and purchased more French wines than German wines when the wine shop played French music. In addition, fast and slow musical rhythms can produce different effects, and consumers give the same food higher ratings when they consume it while listening to pleasant music versus unpleasant music [35,36,37]. Through many studies, it is evident that sound stimuli from multiple aspects, such as background music, noise, and eating sounds, are present in the dining environment. These environmental sounds have specific effects on people and even affect health.

### 2.2. Store Atmosphere and Consumer Emotion Theory

The literature review conducted by Turley and Milliman [15] pointed out that environmental factors, such as lighting, music, temperature, scent, interior design, and tableware are the key elements in creating restaurant ambience. Baker et al. [38] stated that the emotions generated by consumption are often related to the environment of consumption. A favorable consumption situation can be created through the design of a store environment that promotes positive emotions in consumers [39]. Liu and Jang [14] argued that ambience has a significant impact on customer emotions. Grewal et al. [40] pointed out that classical music can promote better perceptions of services and goods. Wilson [41] also demonstrated that classical music can make consumers spend more money on expensive goods. Therefore, the atmospheres created by background music may be a factor that influences consumers’ inferences about merchandise [38]. North et al. [42] suggested that the genre of music is the most influential music-related factor.

**Figure 1:**
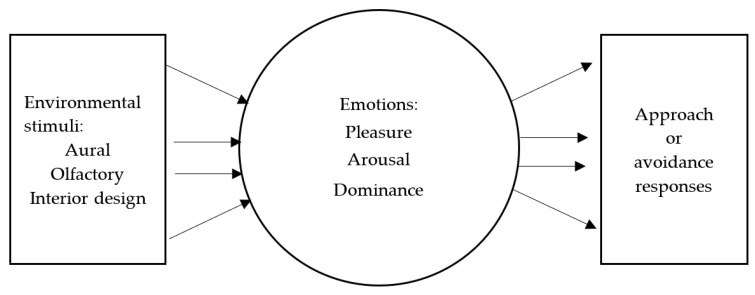
Store atmosphere effect model [8].

As people’s concept of consumption changes, consumers are pursuing not only tangible goods and intangible services, but also an atmospheric experience [13]. All of the sensory feelings that a store induces in customers can be defined as the store’s atmospherics, which is comprised of such elements as color, sound, scent, temperature, and the behaviors of and interactions among people in the store [43,44]. Emotions can also play a key role in many consumers’ behaviors [45]. Yasin et al. [46] pointed out that background music affects consumers’ emotions. Therefore, the creation of a store atmosphere that satisfies consumers is a topic that is relevant to store operators [15]. Restaurant consumers are most sensitive to three factors: the food, the restaurant’s employees, and the restaurant’s atmosphere [47]. Donovan and Rossiter [8] proposed a store atmosphere effect model and pointed out that the store atmosphere may stimulate different emotions in consumers (Figure 1).

The design of the restaurant atmosphere may promote consumption emotions and specific attitudes and behavioral responses in consumers [48]. Chen et al. [49] argued that the construction of a favorable situation and sensory experience can positively impact consumer emotions. Chen and Lee [47] pointed out that the design of the store atmosphere affects consumer responses and purchase intentions. Turley and Milliman [15] showed that the restaurant atmosphere positively affects dining satisfaction. Dragicevic and Rakidzija [50] found that music is an important factor that affects consumers and that happy music can improve customers’ emotions [51]. Therefore, background music will influence pleasure and arousal [52,53,54,55].

In addition, Kotler [56] argued that the creation of a store atmosphere through different environmental designs, can stimulate specific emotions in consumers and affect their purchase intentions and behavior. When there is greater congruence between the music playing and the nature of the establishment, there will be greater congruence between the music and the atmosphere, and consumers will be more likely to enjoy the overall atmosphere and give it higher ratings [57]. Ziv [58] showed that compared to unpleasant background music, playing pleasant background music while consumers dine is more likely to result in higher ratings of food. Therefore, sensory design has a significant impact on consumers [59], and the creation of situations and a good dining experience has a positive impact on consumers’ emotions [60].

### 2.3. Pertinent Literature on Music and Emotion

Kantono et al. [36] showed that people perceive chocolate as tasting sweet when they are listening to music they enjoy and perceive chocolate as tasting bitter when they are listening to music they dislike. Kantono et al. [37] found that music affects the flavor of gelato in the same way. Therefore, the choice of music affects consumers’ dominant perceived taste.

Biswas et al. [10] suggested that high-volume music and noise often increase the excitement levels of consumers, leading to unhealthy food choices; in contrast, low-volume music and noise cause consumers to relax, thereby increasing the sale of healthy foods. Dong et al. [61] showed that higher-pitched music promotes healthier food choices, low-calorie food purchases, and health-promoting activities. The research by Toldos et al. [62] noted that consumers in non-English-speaking countries or regions are more likely to make purchases when English songs are playing in the background. Biswas et al. [10] showed that health food sales increase when low-volume (versus high-volume) music is played in a coffee shop. Kim and Zauberman [63] stated that slow-tempo background music can help alleviate consumers’ dissatisfaction with service delays. Music influences shoppers’ emotions and thus affects their perceptions of time [18]. In short, the emotional impact of music can be positive or negative, and if music is used properly, it can be a competitive tool [64]. High-end restaurants should enhance their customers’ overall dining experience by designing a pleasant atmosphere [65].

A study by Stroebele and de Castro [66] required subjects to keep a detailed food diary for one week under free living conditions, and the results showed that listening to music affected their food intake and resulted in longer meal times; thus, it had an influence on the dining behavior. The results of Mamalaki et al. [67] showed that background music did not affect the intake, meal speed or appetite of young healthy subjects. Therefore, researchers have different opinions on the influence of music on eating behaviors and food intake. Research on music types shows that classical music can increase the enjoyment of food [68]. The research results of Lynar et al. [69] showed that self-selected music is the most effective for inducing a happy state, and classical music with a low arousal is most likely to promote a relaxed state. The research of Spence [70] noted that certain types of sounds and music (under appropriate conditions) can add value and pleasure to the eating experience, while the wrong music can have negative effects. In short, the emotional responses evoked by music can affect multisensory effects, and because emotions have direct and indirect effects on human health, the use of music is an effective strategy for regulating emotions and sensory experiences [23].

Building on the findings above, music atmospherics is a novel concept that not only focuses on auditory sensation but is congruent with other sensations. It can boost the atmosphere and business performance of restaurants. Scozzafava et al. [71] pointed out that service quality has always been considered the most important factor in the experience of restaurant-goers. Accordingly, this study explores and analyses how background music affects consumers’ behavioral responses and emotions. Unlike past studies, this study uses four music conditions—one classical and one jazz arrangement each of a Chinese classical music piece and a Western classical music piece—as the variables to measure the behavioral responses of the study subjects (consumers) and uses an emotional evaluation scale to measure the subjects’ emotional dimensions of pleasure and arousal. In this way, we analyze whether the subjects’ behavioral responses and emotions, during their dining experience, are affected by different music conditions.

Based on the literature described above, the following hypotheses are proposed in this study:

1. The background music preferences of consumers cause significant positive differences in their emotions and behaviors during their dining experience at high-end Chinese restaurants.
2. The background music preferences of consumers can significantly positively impact their emotions and behavioral responses during their dining experience at high-end Chinese restaurants.

## 3. Materials and Methods

### 3.1. Ethical Statement

This study was approved by the Academic Research Ethics Committee of Chinese Culture University. All subjects signed informed consent forms before the study began.

### 3.2. Research Design and Study Subjects

This study combined a factorial experimental design and a survey to collect data through purposive sampling and snowball sampling. The study was conducted during the COVID-19 pandemic from March to June 2020, during which time Taiwan’s restaurants asked consumers not to talk. Patrons of high-end Chinese restaurants in Taipei were selected as the study subjects. The subjects were invited to have dinner at a high-end Chinese restaurant in Taipei in the presence of the background music conditions established for this study and to complete the questionnaires, based on their true feelings regarding the environmental and musical stimuli.

A total of 400 subjects, who volunteered to participate in this study, were recruited and divided into four groups (100 subjects in each group). The four groups were invited to the same high-end Chinese restaurant in Taipei for dinner during four non-overlapping dining periods, one group per dining period, to prevent auditory fatigue from exposure to multiple music conditions in one test. Two classical music pieces, the *Butterfly Lovers Concerto* (Chinese) and the “Canon in D” (Western), were each arranged in two genres, classical and jazz, for this study. The music conditions for groups A, B, C, and D were the *Butterfly Lovers Concerto* (classical) (BL-classical), the *Butterfly Lovers Concerto* (jazz) (BL-jazz), the “Canon in D” (classical) (CD-classical), and the “Canon in D” (jazz) (CD-jazz), respectively. The elements of the musical composition, such as pitch, tempo, and volume, were all edited using Power Sound Editor Free (version 6.9.6), and the volume was maintained at 75 dB. These four music conditions were determined by an expert panel involving three college music professors, three college hospitality management professors, and three high-end restaurant operators.

During the tests, this study provided a consistent scenario. The subjects were invited to eat dinner at the designated high-end Chinese restaurant, which has an elegant atmosphere and stylish surroundings. The professional skills of the restaurant staff aimed to make the subjects feel comfortable. Each group of subjects enjoyed high-quality Chinese cuisine under one of the four music conditions described above. Once the subjects experienced the scenario, they were invited to complete the questionnaires.

A total of 400 questionnaires were distributed and recovered in this study. Once the invalid questionnaires were excluded, 393 valid questionnaires remained (for an effective response rate of 98.25%). The statistical analysis software SPSS 25.0 was used to analyze the results.

#### Recruitment and Testing of the Subjects

Some subjects were recruited via social media (Facebook, Instagram) postings, and snowball sampling was used to recruit elderly subjects who were unfamiliar with social media. Gifts were given to the subjects as an incentive. The subjects had no hearing loss or other hearing-related health problems. Prior to the beginning of the study, all subjects underwent tuning fork tests [72]. According to the test results, none of the subjects had impaired hearing.

### 3.3. Research Tools

#### 3.3.1. Background Music Preference Scale

Table 1 presents the 7-point Likert scale used in this study to evaluate consumers’ background music preferences, based on their feelings towards the classical and jazz arrangements of a Western classical music piece, the “Canon in D”, and a famous Chinese classical music piece, the *Butterfly Lovers Concerto*. The recorded music pieces were performed by professional musicians. Each subject was asked to complete the scale, based on his or her true feelings. The higher the score, the greater the participant’s preference for the music condition; conversely, the lower the score, the lower the participant’s preference for the music condition.

#### 3.3.2. Evaluation Scale for the Emotions during the Dining Experience

Table 2 shows the evaluation scale used to measure emotions during the dining experience. The scale was based on the modified Mehrabian–Russell model from environmental psychology [73] and is scored using a 7-point Likert scale. The subjects were asked to complete the scale, based on their true feelings. The higher the score, the more positive the respondent’s emotions during the dining experience; conversely, the lower the score, the less positive the respondent’s emotions were during the dining experience. In the principal factor analysis, the Kaiser–Meyer–Olkin measure of sampling adequacy reached 0.931, and the chi-squared value of Bartlett’s sphericity test was 2400.486, indicating a statistical significance (*p* < 0.05). Table 2 shows the results of the factor analysis for the scale. For factor 1, pleasure, there were five items with a high load. For factor 2, arousal, there were three items with a high load.

#### 3.3.3. Behavioral Response Scale

Table 3 shows the behavioral response scale, which was adapted from the modified Mehrabian–Russell model from environmental psychology [73]. The subjects were asked to complete the scale, based on their true feelings. Responses were given using a 7-point Likert scale. The higher the score, the more positive the participant’s behavioral response; conversely, the lower the score, the less positive the behavioral response.

#### 3.3.4. Demographic Background Information

It includes two personal background options, such as gender and age.

### 3.4. Data Analysis

This study used two statistical methods, a one-factor analysis of variance (ANOVA) and a multiple regression, to test the research hypotheses. When the one-way ANOVA reached a significant level (*p* < 0.05), the Scheffé post hoc test was used for the post hoc comparisons. Then, in Section 4.7, we use the multiple regression to analyze the predictive power of the different music genres for the consumers’ emotional response and their behavioral responses.

## 4. Results

### 4.1. Demographic Data of the Subjects

#### Gender and Age

Of the 393 subjects included in this study, 148 (37.7%) were male, and 245 (62.3%) were female. There were 124 (31.6%) subjects aged 20–30 years, 97 (24.7%) subjects aged 31–40 years, 89 (22.6%) subjects aged 41–50 years, and 83 (21.2%) subjects aged 51 years and older.

### 4.2. Consumers’ Background Music Preference in the High-End Chinese Restaurants

Among the four music conditions in the high-end Chinese restaurant, the preference for BL-classical was highest, with a mean score of 5.15 points. Overall, the item “I think that the background music is suitable for this restaurant” for BL-classical had the highest single-item score, among all items under all four music conditions (M = 5.45). The item with the second highest score was “I think that the background music in this restaurant is pleasant” for BL-classical (M = 5.40). The item with the lowest score was “I think that the background music is suitable for this restaurant” for CD-jazz (M = 3.13).

### 4.3. Analysis of Consumers’ Emotional Response to the Four Music Conditions during Their Dining Experience

The subjects were divided into four groups in this study, based on the background music conditions in the high-end Chinese restaurant. The levels of agreement between the consumers regarding their emotions during their dining experience, were analyzed using the evaluation scale for emotions during the dining experience. The scale had two dimensions: pleasure and arousal. As the results in Table 4 show, the mean total score (4.94, standard deviation (*SD*) *= 1.64*) was highest for the BL-classical condition. This result indicates that the subjects preferred this music condition. The mean total score (4.00, *SD = 1.74*) for CD-jazz ranked last.

### 4.4. Consumers’ Behavioral Responses to the Music Conditions

Table 5 shows the descriptive statistics for the consumers’ self-reported behavioral responses to the music conditions at the high-end Chinese restaurant. The behavioral response scale had two items, and the means and *SD*s of the total scores and single-item scores were analyzed. The mean score for the consumers’ behavioral responses to the BL-classical music at the high-end Chinese restaurant was highest (M = 4.48), followed by the score for the CD-classical music. Overall, out of the four background music pieces, the subjects believed that the BL-classical music worked best to improve their appetite and increase their purchase intentions.

### 4.5. Differences in Emotions during the Dining Experience under Different Music Conditions

The results of the one-factor ANOVA (Table 6) show that the mean score for the pleasure dimension differed significantly between music conditions (F = 8.609, *p* < 0.01 ***), indicating that the subjects’ degree of pleasure was significantly different under the different music conditions. As the results of the Scheffé post hoc test showed, the mean score on the pleasure dimension for the dining experience with BL-classical (M = 4.74) was significantly higher than for the dining experiences with BL-jazz (M = 4.08) and CD-jazz (M = 4.07). Additionally, the mean score of the pleasure dimension for the dining experience with CD-classical (M = 4.71) was significantly higher than for the dining experience with BL-jazz (M = 4.08) and CD-jazz (M = 4.07). The mean score for the arousal dimension also differed significantly among music conditions (F = 22.417, *p* < 0.01 ***), indicating that the subjects’ degree of arousal was significantly different under different music conditions. As the results of the Scheffé post hoc test showed, the mean score of the arousal dimension for the dining experience with BL-classical (M = 5.08) was significantly higher than for the dining experience with BL-jazz (M = 4.37) and CD-jazz (M = 3.71); the mean score of the arousal dimension for the dining experience with BL-jazz (M = 4.37) was significantly higher than that for the dining experience with CD-jazz (M = 3.71); and the mean score of the arousal dimension was significantly higher for the dining experience with CD-classical (M = 5.11) than for the dining experiences with BL-jazz (M = 4.37) and CD-jazz (M = 3.71). These findings support Hypothesis 1.

### 4.6. Differences in the Behavioral Responses under Different Music Conditions

The results of the one-factor ANOVA, shown in Table 7, show that the consumers’ behavioral responses to different music conditions were significantly different (F = 13.544, *p* < 0.01 ***). As the results of the Scheffé post hoc test showed, the mean score for the behavioral responses to BL-classical (M = 4.57) was significantly higher than the mean score for the behavioral responses to BL-jazz (M = 3.84) and CD-jazz (M = 3.48). Additionally, the mean score for the behavioral response to CD-classical (M = 4.48) was significantly higher than the mean score for the behavioral response to BL-jazz (M = 3.84) and CD-jazz (M = 3.48). These findings also support Hypothesis 1.

### 4.7. Multiple Regression Analysis of the Influences of Consumers’ Emotions during Their Dining Experience on Their Behavioral Responses under Different Background Music Conditions

The multivariate regression analysis was used to examine the influences of consumers’ emotions during their dining experience on their behavioral responses under different background music conditions. The results are shown in Table 8.

I. Under the BL-classical condition, the power of emotions during the dining experience for predicting behavioral responses was 67.7% (R^2^ = 0.677, *p* < 0.001), indicating that consumers’ emotions during their dining experience had a significantly positive impact on their behavioral responses. The pleasure dimension had a greater influence than the arousal dimension on the behavioral responses (t = 3.631, *p* < 0.001).
II. Under the BL-jazz condition, the power of emotions during the dining experience for predicting behavioral responses was 59.1% (R^2^ = 0.591, *p* < 0.001), indicating that consumers’ emotions during their dining experience had a significantly positive impact on their behavioral responses. The pleasure dimension had a greater influence than the arousal dimension on the behavioral responses (t = 2.980, *p* < 0.01).
III. Under the CD-classical condition, the power of emotions during the dining experience for predicting behavioral responses was 41.0% (R^2^ = 0.591, *p* < 0.001), indicating that consumers’ emotions during their dining experience had a significantly positive impact on their behavioral responses. The pleasure dimension had a greater influence than the arousal dimension on the behavioral responses (t = 0.669, *p* < 0.001).
IV. Under the CD-jazz condition, the power of emotions during the dining experience for predicting behavioral responses was 61.3% (R^2^ = 0.613, *p* < 0.001), indicating that consumers’ emotions during their dining experience had a significantly positive impact on their behavioral responses. The arousal dimension had a greater influence than the pleasure dimension on the behavioral responses (t = 5.647, *p* < 0.001).

Taken together, the results of the regression analysis show that consumers’ emotions during their dining experience had a significantly positive impact on their behavioral responses. Therefore, Hypothesis 2 is partially supported.

## 5. Discussion

An excellent restaurant atmosphere can stimulate consumers’ positive emotions during their dining experience and thus lead to positive behavioral responses [10,46,49,74,75], thereby enhancing their dining experience and building consumer satisfaction and loyalty [76,77].

The results of this study show that the BL-classical and CD-classical conditions had a more positive influence on the participants’ emotions during their dining experience than the other two background music conditions did. Consumers’ emotions during a dining experience affect their behavioral responses [2,45,48]. The research location in this study was a high-end Chinese restaurant. The study results show that the BL-classical condition had the most significant positive impact on emotions during the dining experience, followed by the CD-classical condition. It can be inferred that the classical genre is more suitable for high-end Chinese restaurants. Past studies have also suggested that classical music is associated with a perception of a high degree of class and elegance and can enhance the sense of value [9,40,41], which is consistent with the findings of our study. Among the four background music conditions examined in this study, the consumers considered BL-classical the most suitable music for this high-end Chinese restaurant, followed by CD-classical. This result is consistent with the results of past studies: The higher the congruence between the background music and the nature of the establishment, the more positively the background music will impact consumers [18,28,30,57].

Music has always been regarded as a tool for emotion regulation. Many organizations and people use environmental atmosphere to control the use of music, in an effort to adjust or change people’s emotional state [69].

Therefore, we believe that the overall atmosphere of restaurants, including the background music, needs to be carefully designed. The greater the congruence between the background music and the overall restaurant/store atmosphere is, the more positively the background music will influence consumers [11,39,49,62].

## 6. Conclusions

The results of this study show that for the pleasure and arousal dimensions, the influences of the BL-classical and CD-classical music conditions were significantly greater than those of the BL-jazz and CD-jazz conditions, respectively. Additionally, the impact of BL-jazz was significantly greater than that of CD-jazz. These findings confirm that music has a significant impact on emotions during the dining experience [52,54,55,58]. The BL-classical and CD-classical musical selections produced positive emotions in consumers during their dining experience in the high-end Chinese restaurant where this study was conducted.

BL-classical and CD-classical had significantly greater impacts on consumers’ behavioral responses than BL-jazz and CD-jazz. In this way, BL-classical and CD-classical both influenced consumers’ behaviors at the high-end Chinese restaurant. In other words, regardless of the consumers’ music preferences, the presence of background music positively influenced their emotions and behavioral responses during their dining experience. Through the four different music conditions designed in this study, the impact of consumers’ emotions on their behavioral responses during their dining experience could be significantly predicted. In summary, the selection of background music for high-end Chinese restaurants should be based on consumer preferences. However, the perceived effects of background music are independent of individual preferences. The greatest contribution of this study is our prediction of the impact that consumers’ emotions (pleasure and arousal) will have on their behavioral responses during their dining experience, based on our testing of four different music conditions: BL-classical, BL-jazz, CD-classical, and CD-jazz.

## 7. Limitations and Proposals for Future Research

This study conducted a quantitative investigation of background music preferences, emotions during the dining experience, and behavioral responses at a high-end Chinese restaurant in Taipei, Taiwan. Therefore, the results of this study apply only to consumers of high-end Chinese restaurants in Taipei, Taiwan, and they are not applicable to other regions. Restaurant themes, consumers’ personal backgrounds, and consumers’ emotional reactions differ among regions. Therefore, the results of this study can be used as a reference for the selection or planning of background music only in high-end Chinese restaurants in metropolitan areas. Studies that include qualitative interviews are recommended to allow future investigators to gain a better understanding of consumers’ thoughts and to make the research design more comprehensive. Variables, such as other musical elements, including rhythm, timbre, tonality, musical instrument, tempo, track, and genre, can be introduced to enrich the research on the background music in retail settings.

## Figures and Tables

**Table 1:** Background music preference scale.

Dimension	Measurement Dimension	Measurement Method
Background music preference	Preference	I truly like this piece of music.
		The background music is suitable for this restaurant.
		The background music in this restaurant is pleasant.
		The background music in this restaurant can improve the service quality.
		The background music in this restaurant makes the food look more delicious.
		I think that the background music in this restaurant can enhance the aesthetic experience.

**Table 2:** Factor analysis of the evaluation scale for the emotions during the dining experience.

Item	Factor 1 Pleasure	Factor 2 Arousal
The background music makes me feel refreshed.	0.892	
The background music makes me feel comfortable.	0.937	
The background music is elegant.	0.920	
The background music makes me feel relaxed.	0.900	
The background music enhances the perceived value of the restaurant.	0.886	
The background music makes me feel energetic.		0.726
The background music makes me feel excited.		0.899
The background music is dynamic.		0.617
Eigenvalue	1.098	5.185
Explanatory variance (%)	13.72	64.81
Cumulative variance (%)	78.53	64.81
Kaiser–Meyer–Olkin measure of the sampling adequacy		0.931
Cronbach’s α	0.886	0.855

**Table 3:** Behavioral response scale.

Dimension	
Behavioral response	I think that the background music in this restaurant can increase my appetite.
	I think that the background music in this restaurant can increase my purchase intentions.

**Table 4:** Descriptive analysis of the consumers’ emotional responses to different music conditions during their dining experience (*n* = 393).

Dimension	BL-Classical	Mean Score (M)	Standard Deviation (SD)	Rank
Emotions during the dining experience		4.94	1.64	1
Dimension 1: pleasure		4.84	1.56	
Dimension 2: arousal		5.11	1.49	
Dimension	BL-jazz	Mean score (M)	Standard deviation (SD)	Rank
Emotions during the dining experience		4.13	1.60	3
Dimension 1: pleasure		3.99	1.58	
Dimension 2: arousal		4.38	1.59	
Dimension	CD-classical	Mean score (M)	Standard deviation (SD)	Rank
Emotions during the dining experience		4.86	1.51	2
Dimension 1: pleasure		4.73	1.53	
Dimension 2: arousal		5.08	1.44	
Dimension	CD-jazz	Mean score (M)	Standard deviation (SD)	Rank
Emotions during the dining experience		4.00	1.74	4
Dimension 1: pleasure		4.17	1.73	
Dimension 2: arousal		3.71	1.72	

Note: A 7-point Likert scale was used for which: 1 = strongly disagree, 2 = disagree, 3 = somewhat disagree, 4 = neutral, 5 = somewhat agree, 6 = agree, and 7 = strongly agree.

**Table 5:** Analysis of the consumers’ behavioral responses to the different music conditions (*n* = 393).

BL-Classical	Mean (M)	Standard Deviation (SD)	Rank
Behavioral response	4.48	1.43	1
The background music in this restaurant can increase my appetite.	4.51	1.50	
The background music in this restaurant can increase my purchase intentions.	4.45	1.36	
BL-jazz	Mean (M)	Standard deviation (SD)	Rank
Behavioral response	3.84	1.46	3
The background music in this restaurant can increase my appetite.	3.83	1.48	
The background music in this restaurant can increase my purchase intentions.	3.85	1.45	
CD-classical	Mean (M)	Standard deviation (SD)	Rank
Behavioral response	4.40	1.42	2
The background music in this restaurant can increase my appetite.	4.26	1.47	
The background music in this restaurant can increase my purchase intentions.	4.53	1.37	
CD-jazz	Mean (M)	Standard deviation (SD)	Rank
Behavioral response	3.49	1.67	4
The background music in this restaurant can my increase appetite.	3.56	1.72	
The background music in this restaurant can increase my purchase intentions.	3.41	1.63	

**Table 6:** One-factor ANOVA of the emotions during the dining experience under different music conditions (*n* = 393).

Dependent Variable	Music Condition	Number of Samples (n)	Mean Score (M)	Standard Deviation (SD)	F Value (F)	Scheffé
Pleasure	BL-classical	99	4.74	1.20	8.609 ***	(1) > (2), (4); (3) > (2), (4);
	BL-jazz	97	4.08	1.23		
	CD-classical	98	4.71	1.27		
	CD-jazz	99	4.07	1.36		
Arousal	BL-classical	99	5.08	1.25	22.417 ***	(1) > (2), (4); (2) > (4); (3) > (2), (4);
	BL-jazz	97	4.37	1.38		
	CD-classical	98	5.11	1.34		
	CD-jazz	99	3.71	1.56		

Note: 1. *** *p* < 0.001; 2. (1) BL-classical, (2) BL-jazz, (3) CD-classical, and (4) CD-jazz.

**Table 7:** One-factor ANOVA of the behavioral responses to different musical conditions (*n* = 393).

Dependent Variable	Music Condition	Number of Samples (n)	Mean Score (M)	Standard Deviation (SD)	F Value (F)	Scheffé
Behavioral response	BL-classical	99	4.57	1.33	13.544 ***	(1) > (2), (4); (3) > (2), (4);
	BL-jazz	97	3.84	1.34		
	CD-classical	98	4.48	1.34		
	CD-jazz	99	3.48	1.59		

Note: 1. *** *p < 0.001; 2.* (1) BL-classical, (2) BL-jazz, (3) CD-classical, and (4) CD-jazz.

**Table 8:** Results of the multiple regression analysis of the influence of consumers’ emotions during their dining experience on their behavioral responses under different background music conditions (*n* = 393).

	BL-Classical		BL-Jazz		CD-Classical		CD-Jazz	
	*β*	*t*	*β*	*t*	*β*	*t*	*β*	*t*
Pleasure	0.432	3.631 ***	0.443	2.980 **	0.653	3.882 ***	0.630	0.490
Arousal	0.419	3.525 **	0.346	2.323 *	−0.014	−0.084	0.728	5.647 ***
R^2^	0.677		0.591		0.410		0.613	
F	100.775 ***		6.834 ***		33.369 ***		75.314 ***	

Note: * *p* < 0.05, ** *p* < 0.01, and *** *p* < 0.001.

## Data Availability

Not applicable.
